# Carbohydrate NMR chemical shift prediction by GeqShift employing E(3) equivariant graph neural networks

**DOI:** 10.1039/d4ra03428g

**Published:** 2024-08-22

**Authors:** Maria Bånkestad, Kevin M. Dorst, Göran Widmalm, Jerk Rönnols

**Affiliations:** a Department of Information Technology, Uppsala University Sweden maria.bankestad@it.uu.se; b RISE Research Institutes of Sweden Stockholm Sweden; c Department of Organic Chemistry, Stockholm University Sweden

## Abstract

Carbohydrates, vital components of biological systems, are well-known for their structural diversity. Nuclear Magnetic Resonance (NMR) spectroscopy plays a crucial role in understanding their intricate molecular arrangements and is essential in assessing and verifying the molecular structure of organic molecules. An important part of this process is to predict the NMR chemical shift from the molecular structure. This work introduces a novel approach that leverages E(3) equivariant graph neural networks to predict carbohydrate NMR spectral data. Notably, our model achieves a substantial reduction in mean absolute error, up to threefold, compared to traditional models that rely solely on two-dimensional molecular structure. Even with limited data, the model excels, highlighting its robustness and generalization capabilities. The model is dubbed *GeqShift* (geometric equivariant shift) and uses equivariant graph self-attention layers to learn about NMR chemical shifts, in particular since stereochemical arrangements in carbohydrate molecules are characteristics of their structures.

## Abstract 2

Visual abstract of GeqShift, an E(3) equivariant graph neural network for predicting carbohydrate NMR shifts. The model excels in stereochemical invariance, offering superior molecular geometry understanding over traditional methods.

## Graphical abstract

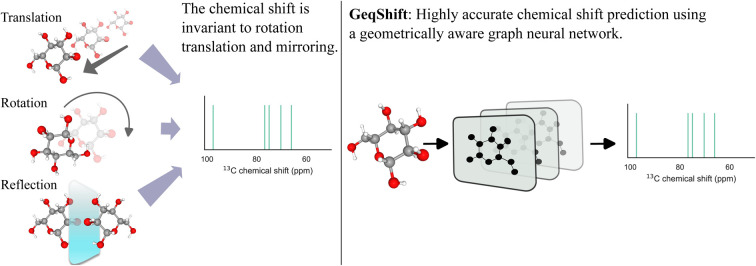

## 1 Introduction

Carbohydrates are intricate organic compounds that ubiquitously occur in all living organisms. Their significance spans across all domains of life, but especially in cell–cell interactions and disease processes. In recent decades, a remarkable advancement in our comprehension of carbohydrate chemistry and biology has been attributed to their vital importance. The molecular structure of carbohydrates is notably complex and diverse and, therefore, challenging for chemists to construct and manipulate.^1,2^ The role of carbohydrates in biological processes heavily depends on their three-dimensional structures, which include the covalent bonds and the conformations these molecules adopt over time. Nuclear magnetic resonance (NMR) spectroscopy is a fundamental technique to decipher the intricate three-dimensional structure of molecules. This study introduces a cutting-edge machine-learning model to interpret NMR spectra, which considers molecule geometries and known symmetries.

The inherent complexity of carbohydrate molecules in structural studies and stereochemical assignments stems from two key factors: their large number of stereocenters and the extensive possibilities for interconnecting monosaccharides. For example, combining two glucopyranosyl residues can yield as many as 19 distinct disaccharides, each with a unique structure.^3^ Additionally, variations in substitution patterns, like acetylation and sulfonation, further contribute to the complexity of carbohydrate structures. Determining carbohydrate structures by NMR spectroscopy can be a formidable task.^4^

**Fig. 1:**
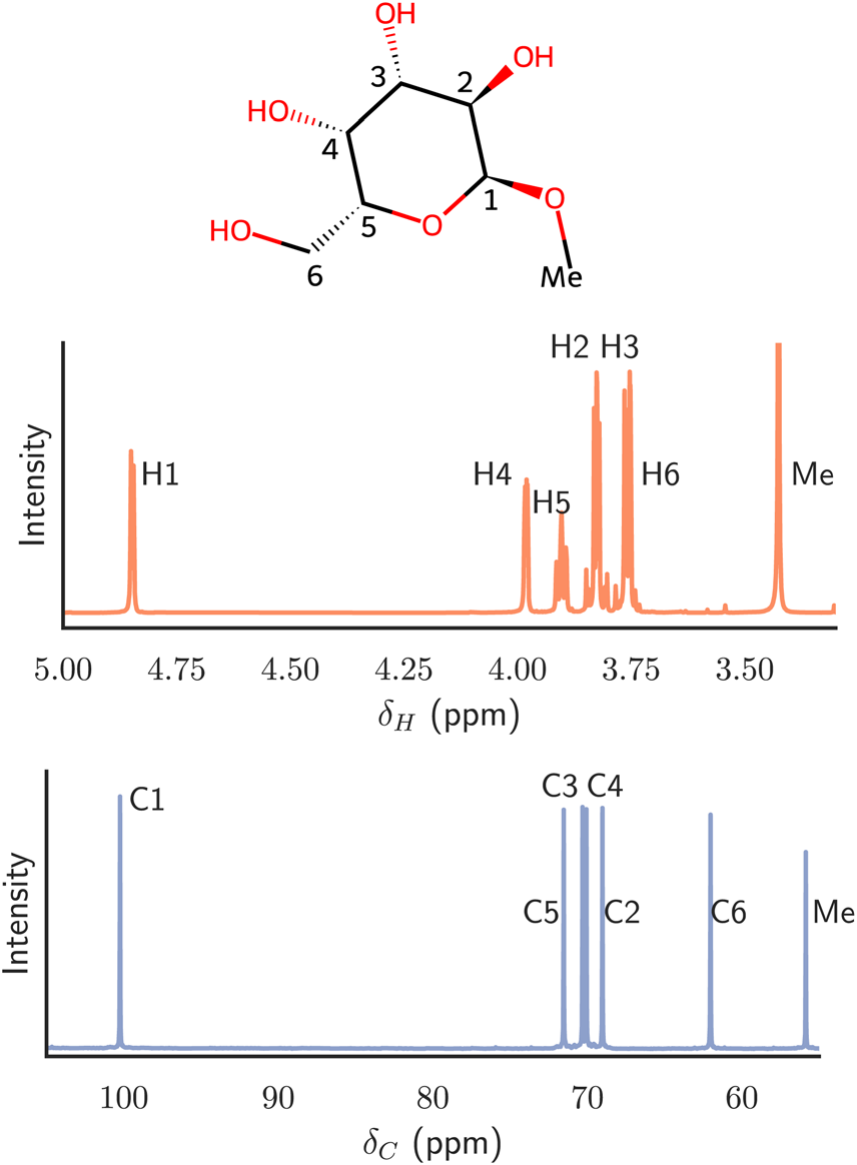
Schematic representation of methyl α-d-galactopyranoside and ^1^H and ^13^C NMR spectra thereof. The peaks of the specific protons (from H1 to H6 and the *O*-methyl group) and the corresponding carbons are indicated in the plots. Resonances are annotated (H1–H6, Me; C1–C6, Me) close to their chemical shifts.

The peaks observed in an NMR spectrum of a molecule provide valuable information about the presence of nuclei and their chemical surroundings, such as carbon and hydrogen isotopes ^13^C and ^1^H, and how they are interconnected. Fig. 1 provides examples of ^13^C and ^1^H NMR spectra for a monosaccharide.

**Fig. 2:**
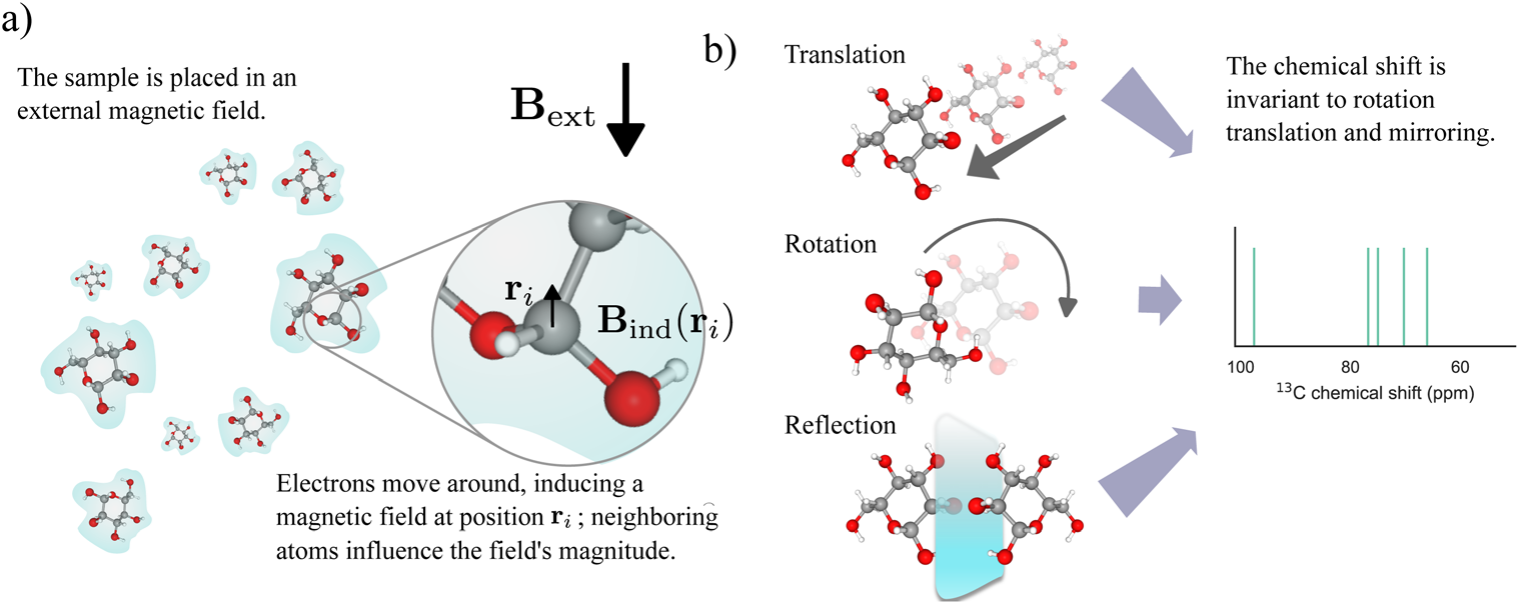
(a) The compound under examination moves within a fluid environment and interacts with an external magnetic field denoted as **B**_ext_. An induced magnetic field **B**_ind_(**r**_*i*_) at a specific position **r**_*i*_ determines the chemical shift of a resonating nucleus. (b) The chemical shift *δ* remains constant under the Euclidean group E(3), *i.e.*, it is unaffected by translation, rotation, and reflection.

The position of a peak for a particular nucleus, indicated by its chemical shift *δ* (*δ*_H_ and *δ*_C_ for ^1^H and ^13^C chemical shifts, respectively), corresponds to the resonance frequency of the nucleus within a magnetic field. The local environment of the atom, especially the electron density in the vicinity of the nucleus, strongly influences this resonance frequency (see Fig. 2). Besides the atomic species of the studied nucleus, the primary factors influencing chemical shifts are the neighboring covalently bonded atoms within the molecule because the electronegativity of these nearby atoms correlates closely with the observed chemical shifts. Electron-withdrawing groups, like oxygen and fluorine, located near the observed nuclei deshield them, increasing their chemical shifts. Conversely, proximity to electron-donating groups enhances shielding, thereby decreasing the chemical shifts.

**Fig. 3:**
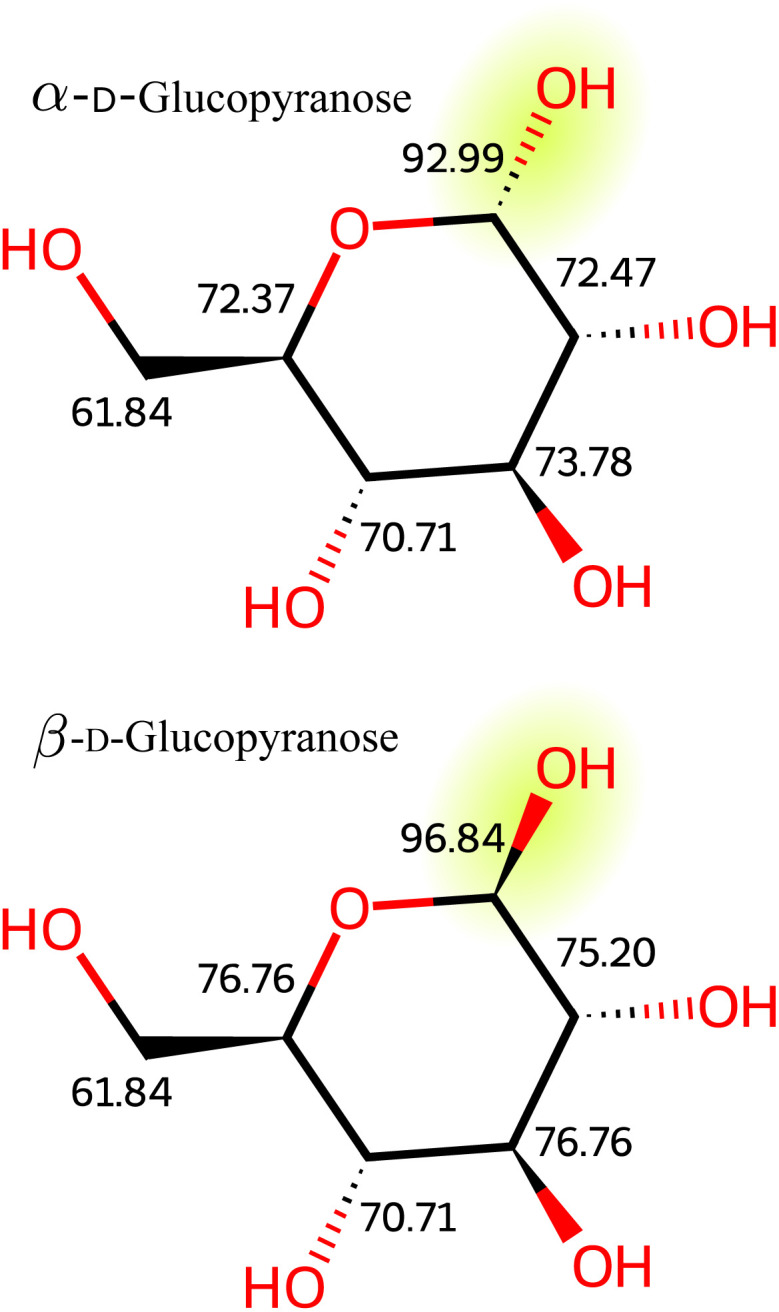
^13^C NMR chemical shifts of two glucose isomers, α-d-glucopyranose and β-d-glucopyranose. These isomers differ only in the stereochemistry of the anomeric center (highlighted). This subtle variation substantially impacts the chemical shifts in an NMR spectrum.

In molecular ring systems (appearing in carbohydrates), the orientation of a hydrogen atom, either axially or equatorially, significantly impacts its *δ*_H_ value. Similarly, for carbon nuclei in a ring system, the arrangement of substituents they carry influences their *δ*_C_ value. Fig. 3, showing the ^13^C chemical shifts of α- and β-glucopyranose, illustrates this discrepancy. The change in configuration at the anomeric center not only affects the chemical shift of highlighted anomeric carbon but also has a ripple effect, altering the shifts of all carbon atoms in the molecule. It is important to note that spatial interactions can influence chemical shifts beyond the effects of covalent bonds.^5^

A standard method for predicting the chemical shifts of carbohydrate molecules involves utilizing an extensive database of known carbohydrates.^6^ This approach entails comparing new carbohydrate structures with those existing in the database, making necessary adjustments for recognized patterns around glycosidic bonds.

The CASPER program^7^ relies on a relatively small set of NMR data of glycans. It uses approximations to predict chemical shifts of glycan structures not present in the database, which facilitates the coverage of a large number of structures. However, the reliance on these databases is less effective when new structures containing previously uncharacterized sugar residues are encountered.

Alternatively, chemical shifts can be estimated using Quantum Mechanical Density Functional Theory (DFT) calculations.^8^ While this technique is effective for many molecules, it comes with substantial computational demands, making it both costly and time-consuming. A notable advancement in carbohydrate chemical shift calculation was recently published by Palivec *et al.*^9^ and involves an in-depth simulation of the water environment surrounding the molecules under study. This approach employs molecular dynamics and DFT to calculate chemical shifts for small carbohydrate molecules, including mono-, di-, and one trisaccharide.

As previously mentioned, the relationship between a molecule and its chemical shift is intricate, suggesting the utility of artificial neural networks (ANNs), recognized as universal approximators, to model this relationship from data. Neural networks, a subset of machine learning methods, are adept at learning high-dimensional feature spaces and capturing subtle, intricate patterns within the data.^10^ For predicting chemical shifts, neural networks trained on carefully constructed datasets of experimental chemical shifts can account for various influencing factors, such as electronic environments, steric effects, and long-range interactions, leading to fast, accurate, and reliable chemical shift predictions. As early as 1991, Meyer *et al.*^11^ proposed using a feed-forward network to identify ^1^H NMR spectra for oligosaccharides. More recently, graph neural networks (GNNs) have emerged to predict chemical shifts.^12^ Some of these models use only the molecular structure (the atoms and their bonds) as input,^13–15^ while others incorporate atom–atom pairwise distances as additional input features.^16,17^

While these models demonstrate strong performance for numerous molecules, they struggle when dealing with molecules featuring complex stereochemistry, such as carbohydrates. It is appropriate to assume that these molecules must be treated as dynamic, three-dimensional entities for accurate representation, demanding a network capable of capturing this complexity. This study proposes a model that integrates the three-dimensional molecular structure while preserving the fundamental symmetries of the underlying physics of the molecule.

More specifically, we introduce an E(3) equivariant graph neural network, also known as an Euclidean neural network.^18^ Equivariance is a transformation property that assures a consistent response when a feature transforms. An example of equivariance is the intramolecular forces holding the atoms together in a molecule. These forces are equivariant to rotation since these forces rotate together with the molecule. An equivariant function preserves relationships between input (molecule) and output (interatomic forces) during transformations. If we have an equivariant function deriving the interatomic forces, these derived forces rotate with the molecule.

An Euclidean neural network is equivariant to the Euclidean group E(3), which is the group of transformations in the Euclidean space, including rotation, translation, and mirroring. Compared to a network that solely considers pairwise distance, an equivariant network considers the relative distance between atoms, encompassing both pairwise distance and pairwise direction. Euclidean neural networks have recently gained recognition for their success in various chemistry applications, spanning from modeling molecule potential energy surfaces^19^ to predicting toxicity^20^ and studying protein folding.^21^

Our model, denoted as *GeqShift* (geometric equivariant shift), is a GNN that utilizes equivariant graph self-attention layers^22^ to learn chemical shifts, particularly when stereochemistry is crucial. These attention layers update the node features by considering features of close nodes, so-called neighbors, and weights these neighbors to emphasize the most important information, using so-called attention weights. Our contribution is three-fold: the chemical shift prediction model GeqShift, an innovative data augmentation method inspired by the dynamic movement of molecules in a fluid, and a compiled carbohydrate chemical shift dataset suitable for machine learning applications. By making this dataset public, we hope to stimulate further research in data-driven automated chemical shift analysis.

Our experiments demonstrate that our model and training approach achieve precise predictions, especially in intricate stereochemistry cases. Notably, for the carbohydrate dataset, our network reaches mean absolute errors (MAEs) of 0.31 for *δ*_C_ and 0.032 for *δ*_H_.

## 2 Results

Our model is trained on ^13^C and ^1^H NMR chemical shift data from the CASPER program,^7^ which is further detailed in the methods section. We evaluate the model's generalization capability using cross-validation. In machine learning, the fundamental assumption is that data points are independently and identically distributed (iid) samples from a specific distribution, such as a distribution of carbohydrates. Validation shows that the model generalizes well to other samples from the same distribution, indicating its ability to interpolate between data points.^23^ However, it is important to note that there are no general guarantees for performance on data from different distributions. Tenfold cross-validation is a well-established validation method, where 10% of the data is withheld during training and used for testing, repeated ten times with different subsets.^24^ This ensures that each carbohydrate sample is tested on a model that has not seen that specific carbohydrate before, providing a robust measure of the model's generalization capabilities within the given distribution. We let each split maintain a balanced mono-, di-, and trisaccharides distribution. Each split comprises approximately 336 carbohydrate structures for training and 39 for testing.

A molecule is inherently dynamic, continuously changing its conformation. The likelihood of these conformations follows the Boltzmann distribution, *p*(**R**) ∼ exp(−*E*(**R**)), where *E* is the molecule energy function and **R** its conformation. Conventionally, in data-driven models, this problem is alleviated by selecting the conformation with the lowest energy, implying the highest probability. This is typically determined through methods like density functional theory (DFT) simulation.

We take a different approach by considering the molecule conformation as dynamic, with not just one but an ensemble of conformations. The predicted NMR chemical shift varies depending on the conformation, resulting in an ensemble of predictions per molecule. The final prediction is the average. We use this technique during both training and testing.

**Fig. 4:**
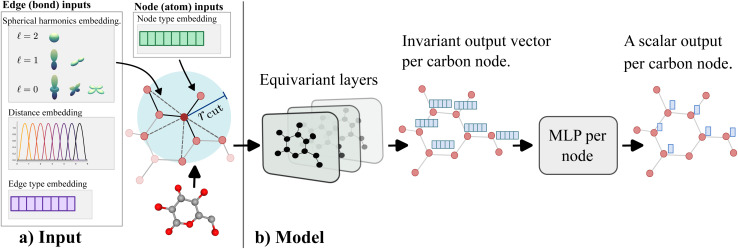
An overview of the model. The left side (labeled a) shows the components involved in processing molecule input data. These include node embeddings with atom type and neighboring hydrogen information and edge embeddings representing bond types and relative distances between connected nodes. The *r*_cut_ parameter denotes the cutoff radius for defining neighboring atoms. The model architecture is illustrated on the right side (labeled b). It consists of *K* equivariant layers, with the final layer producing an invariant vector for each node. Nodes containing chemical shift data are processed individually, passing through a multi-layer perceptron (MLP) to generate an invariant chemical shift prediction.

In machine learning terms, this is a data augmentation technique. We hypothesize that this will enhance the generalization capacity of the model, especially given the limited size of the training dataset. As a result, our final model, GeqShift, does not rely on a specific low-energy conformation as input, enabling effective generalization to molecules not seen during training. Fig. 4 presents an overview of the model.

To establish a baseline, we compare our model with the scalable GNN by Han *et al.*,^15^ referred to as SG-IMP-IR, which performs state-of-the-art results on the NMRShiftDB2 dataset.^25^ Additionally, we conducted six ablations to assess the effectiveness of various components in our model, as summarized in Table 1. These evaluations include comparing the use of an invariant version (inv) of the model, the same as setting 
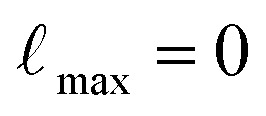
, the maximum degree of the irreducible representations of the hidden layers (explained further in Section 4.1). Furthermore, we examined the impact of testing and training on an ensemble of conformations by evaluating the model on only a single conformation (1T) and training and testing on a single conformation (1TT). It is important to note that the train/test splits are consistent across all models, with data augmentation achieved by sampling multiple conformations per molecule.

**Table:** An overview of our two models with their training and test data variations

Models	Nbr conf. train	Nbr conf. test	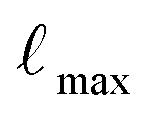 in hidden layers
GeqShift_1TT_inv	1	1	0
GeqShift_1TT	1	1	2
GeqShift_1T_inv	100	1	0
GeqShift_1T	100	1	2
GeqShift_inv	100	100	0
GeqShift	100	100	2

**Fig. 5:**
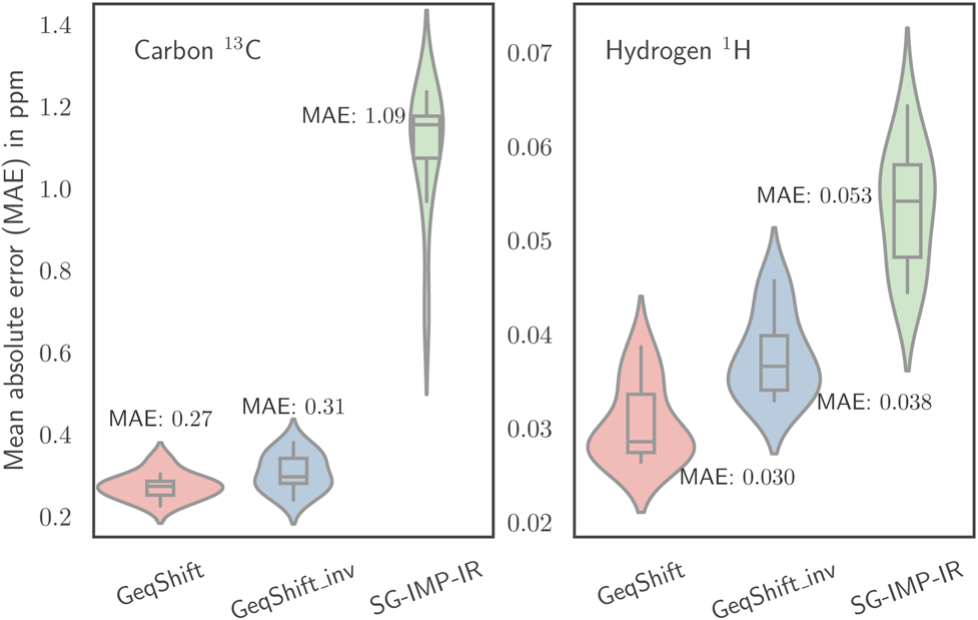
Comparison of the test prediction accuracy in mean absolute error MAE between the baseline model SG-IMP-IR and our proposed model GeqShift, and its invariant version GeqShift_inv. The result is visualized using violin plots.

Fig. 5 presents an overview of the performance of the model using violin plots, a combination of a box plot, and a density plot.^26^ Furthermore, Table 2 provides a detailed comparison of the models, emphasizing prediction accuracy for different types of carbohydrates, including mono-, di-, and trisaccharides.

Comparison of prediction test accuracy for ^13^C and ^1^H chemical shifts in terms of MAE (ppm) and RMSE (ppm) split between monosaccharides, disaccharides, and trisaccharides. The accuracy is presented as the ten-fold mean, standard deviation in parenthesis. SG-IMP-IR refers to a state-of-the-art model^15^ retrained with our data. All GeqShift models were produced in this work. Details of how the simulation tools, carbohydrate structure database (CSDB), and NMR database (NMRDB) predictions are found in Section 4.3

**Table:** 

	Monosaccharides ^13^C		Disaccharides ^13^C		Trisaccharides ^13^C	
	MAE	RMSE	MAE	RMSE	MAE	RMSE
CSDB	1.23 (1.00)	3.40 (3.94)				
NMRDB	2.02 (0.29)	2.87 (0.41)				
SG-IMP-IR	1.18 (0.20)	1.61 (0.30)	1.02 (0.17)	1.53 (0.37)	1.13 (0.16)	1.61 (0.20)
GeqShift_1 TT_inv	0.54 (0.12)	0.86 (0.23)	0.44 (0.07)	0.73 (0.15)	0.65 (0.11)	1.06 (0.21)
GeqShift_1 TT	0.55 (0.15)	0.90 (0.37)	0.47 (0.08)	0.75 (0.17)	0.63 (0.11)	1.05 (0.24)
GeqShift_1T_inv	0.39 (0.11)	0.69 (0.23)	0.28 (0.06)	0.51 (0.16)	0.37 (0.10)	0.64 (0.21)
GeqShift_1T	0.34 (0.08)	0.61 (0.19)	0.25 (0.06)	0.48 (0.18)	0.33 (0.09)	0.57 (0.20)
GeqShift_inv	0.37 (0.11)	0.66 (0.23)	0.26 (0.06)	0.49 (0.16)	0.33 (0.08)	0.59 (0.14)
GeqShift	**0.31** (0.08)	**0.58** (0.18)	**0.23** (0.06)	**0.46** (0.19)	**0.30** (0.09)	**0.53** (0.16)

**Table:** 

	Monosaccharides ^1^H		Disaccharides ^1^H		Trisaccharides ^1^H	
	MAE	RMSE	MAE	RMSE	MAE	RMSE
CSDB	0.11 (0.032)	0.19 (0.083)				
NMRDB	0.30 (0.036)	0.37 (0.039)				
SG-IMP-IR	0.071 (0.026)	0.110 (0.039)	0.045 (0.007)	0.075 (0.014)	0.055 (0.011)	0.087 (0.020)
GeqShift_1 TT_inv	0.064 (0.011)	0.100 (0.022)	0.049 (0.009)	0.076 (0.017)	0.067 (0.009)	0.102 (0.015)
GeqShift_1 TT	0.061 (0.016)	0.115 (0.053)	0.041 (0.006)	0.061 (0.012)	0.060 (0.010)	0.103 (0.030)
GeqShift_1T_inv	0.046 (0.014)	0.078 (0.040)	0.034 (0.006)	0.053 (0.012)	0.050 (0.010)	0.079 (0.018)
GeqShift_1T	0.037 (0.009)	0.062 (0.020)	0.028 (0.003)	0.046 (0.010)	0.038 (0.009)	0.057 (0.017)
GeqShift_inv	0.044 (0.015)	0.077 (0.041)	0.030 (0.004)	0.048 (0.011)	0.043 (0.009)	0.069 (0.015)
GeqShift	**0.035** (0.009)	**0.057** (0.018)	**0.026** (0.003)	**0.044** (0.011)	**0.033** (0.009)	**0.052** (0.016)

Among our models, GeqShift emerges as the top-performing model, closely followed by GeqShift_inv. Compared to using just one conformation per molecule for training, we observe a significant performance improvement when using an ensemble of 100 conformations. For instance, in the case of monosaccharides, the mean absolute error (MAE) notably decreases from 0.55 to 0.37 when trained with 100 conformations. Subsequently, it further drops slightly to 0.31 when also predicting 100 conformations. These results underscore the advantage of incorporating multiple conformations in our training and prediction processes.

GeqShift surpasses the CSDB and NMRDB simulation tools in predicting carbon and proton chemical shifts. Although this comparison is not entirely straightforward, since the CSDB database contains molecules that are part of the testing distribution but does not include all molecules from the training dataset, it still highlights GeqShift's superior generalization capability.

**Fig. 6:**
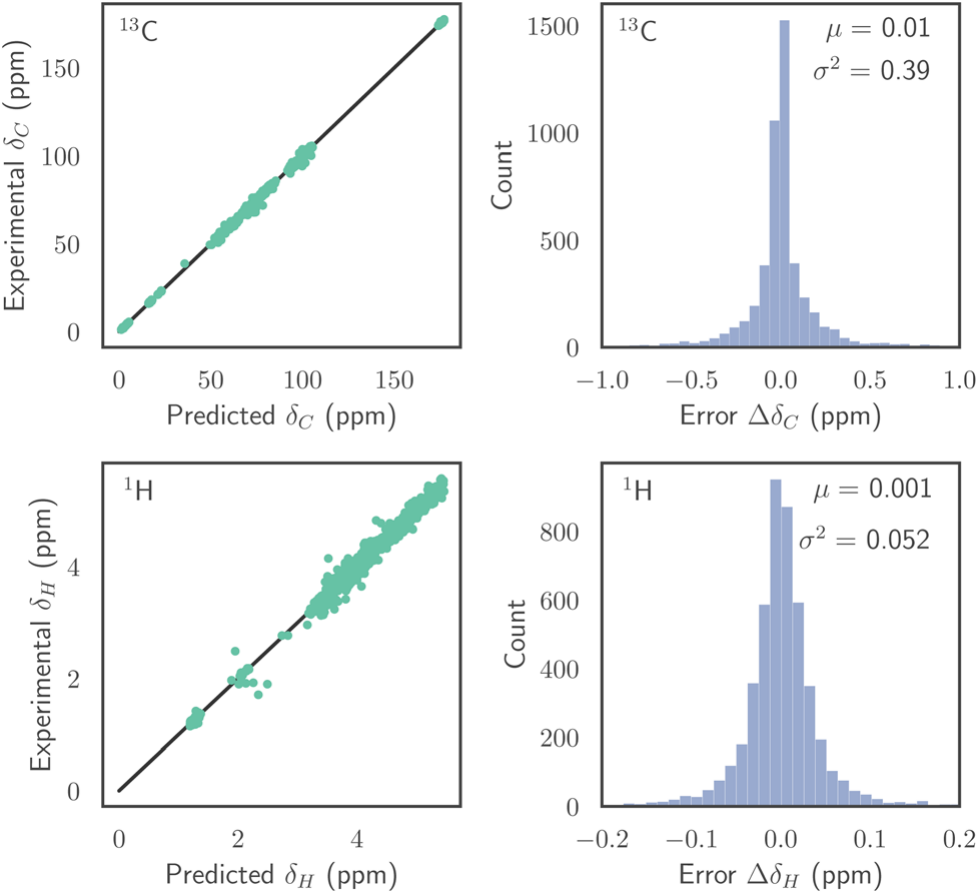
The figure examines the test prediction outcomes of our proposed method, GeqShift. To the left, scatter plots illustrate the relationship between actual and predicted values. Histograms representing the distribution of prediction errors Δ*δ* are shown on the right.

In Fig. 6, we delve deeper into the prediction accuracy of our best-performing method, GeqShift. The figures within this plot illustrate histograms of prediction errors and scatter plots depicting the relationship between the actual and predicted values for both ^13^C and ^1^H nuclei. We combined the test sets' prediction results across all ten cross-validation folds to create these visualizations. Notably, the distributions of prediction errors are approximately zero-centered, with a standard deviation of 0.39 for ^13^C and 0.052 for ^1^H.

**Fig. 7:**
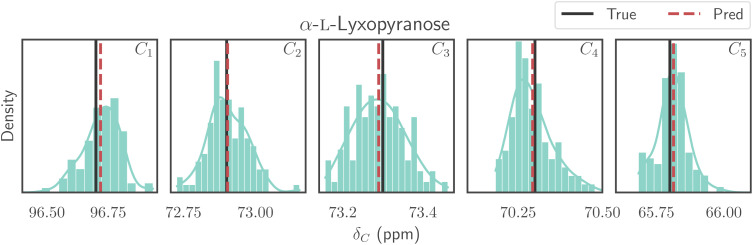
A histogram representing the test predictions of ^13^C chemical shifts obtained from 100 different molecular geometries of the monosaccharide α-l-lyxopyranose. We highlight the prediction mean and the actual peak value. While various geometries yield slightly different chemical shift values, the average of these peaks closely approximates the experimentally determined value.

Fig. 7 visualizes the predictions from the whole ensemble of conformations for the monosaccharide α-l-lyxopyranose. The figure displays histograms representing the predictions for each ^13^C atom in the molecule, the ensemble mean, and the actual NMR peaks. These histograms showcase the distribution of predicted values, allowing for a comparison with a real NMR spectrum (refer to Fig. 1). Furthermore, the ensemble of predictions per chemical shift enables an estimation of prediction uncertainty by examining the standard deviation.

### 2.1 Out of distribution predictions

In the previous section, we examined the model's ability to generalize to other molecules within the same distribution as the training data using cross-validation. Now, we focus on evaluating the model's capability to generalize beyond the training data distribution. To achieve this, we omit specific molecular structures from the training dataset and assess whether the model can accurately predict the NMR spectrum for these excluded structures. This approach serves as a stress test for the model's robustness and extrapolation abilities. Table 3 lists the excluded substructures used as the test set for this evaluation.

**Table:** Description of the excluded structures: these molecular structures were deliberately omitted from the training data and subsequently used as a test set to evaluate the model's performance

Name	Structures left out	Nbr remove
Xyl	All with a Xyl residue	10
Qui	All with a Qui residue	7
Ur_acid	All with a uronic acid GlcA, sManA and GalA left out	14
Ur_acid/GlcA	All with a uronic acid but keep GlcA (ManA and GalA left out)	8
Ac	Remove all with acetylated compounds	19
34Ac	Remove all with acetylated compounds at carbon 3 and 4	10

**Fig. 8:**
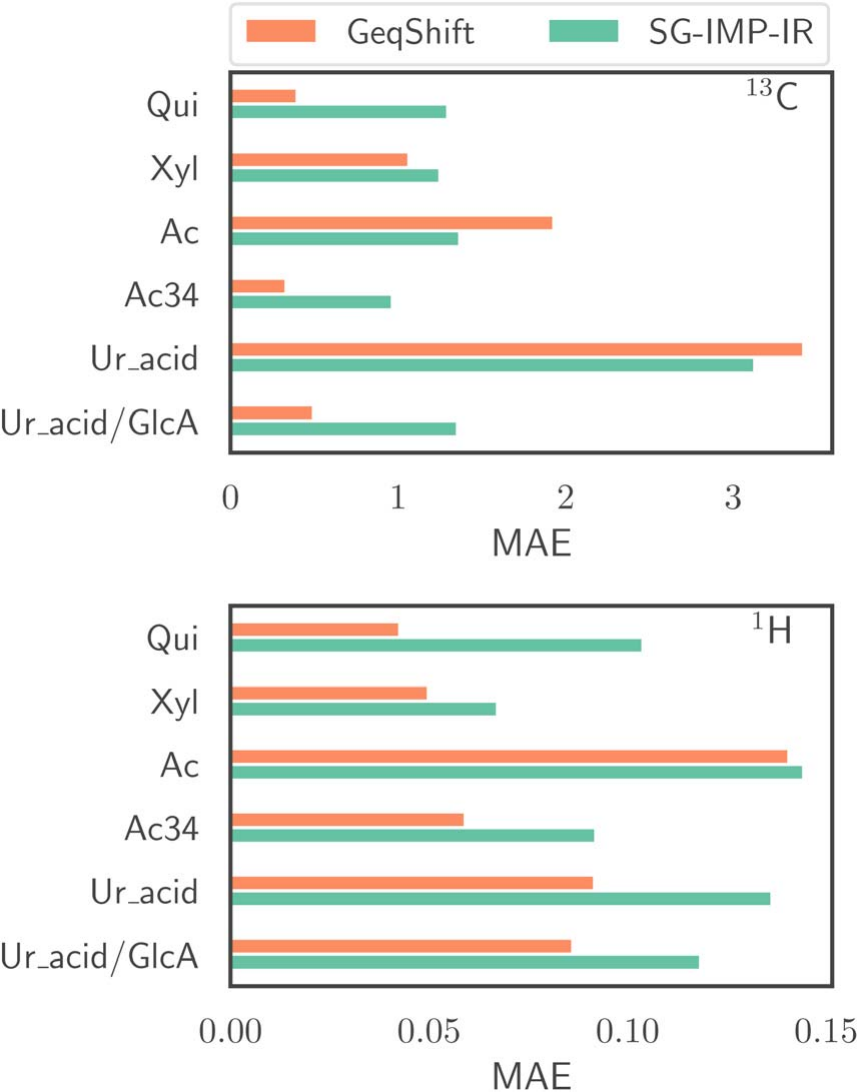
Prediction performance for chemical shifts (^13^C and ^1^H) in terms of mean absolute error (MAE) for the out-of-distribution evaluation. The specific structures that were excluded from the training data and then used as a test set are listed in Table 3.

Fig. 8 compares the prediction accuracy of GeqShift with SG-IMP-IR, where GeqShift outperforms SGIMPIR on a majority of the substructures. This experiment underscores the importance of including structurally similar molecules in the training data for accurate machine learning predictions. Specifically, when the model is trained on the Ur_acid dataset with all uronic acids excluded, it performs poorly in predicting the NMR spectra of molecules containing uronic acids. However, when GlcA, a specific uronic acid, is included in the training data, the model's performance significantly improves for the excluded uronic acid molecules, ManA and GalA. This result suggests that similar structural motifs in the training data enhance the model's ability to generalize to new, unseen molecules within the same chemical family. Furthermore, it demonstrates the model's capability to extrapolate structural information from one molecule (GlcA) to different but related molecules (ManA and GalA).

### 2.2 Polysaccharides

**Fig. 9:**
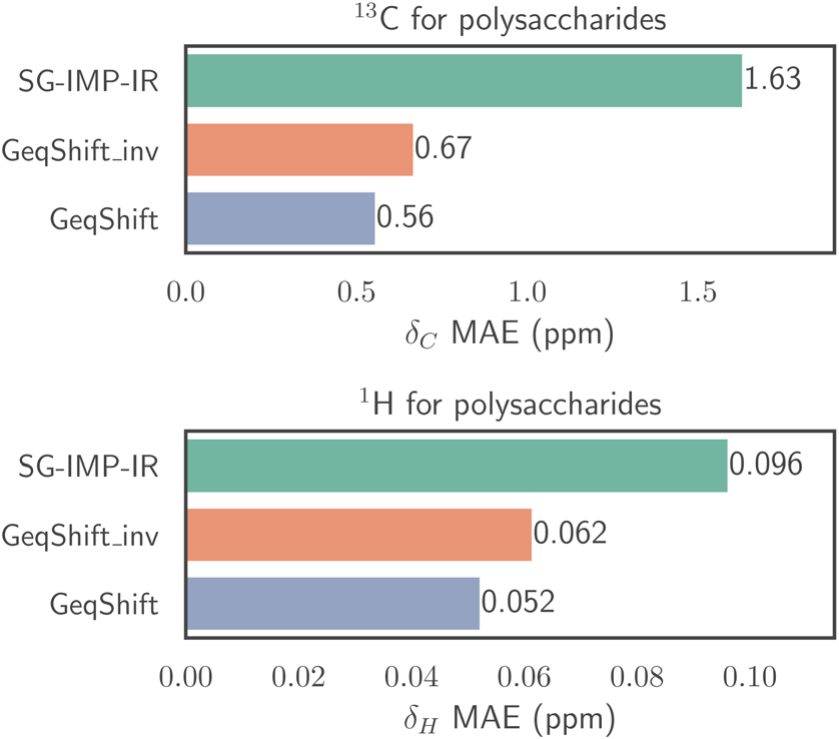
Prediction performance for chemical shifts (^13^C and ^1^H) in terms of mean absolute error (MAE) within the context of the two polysaccharides introduced in Fig. 10. In this evaluation, the models employ an average prediction derived from the ten models trained during ten-fold cross-validation.

**Fig. 10:**
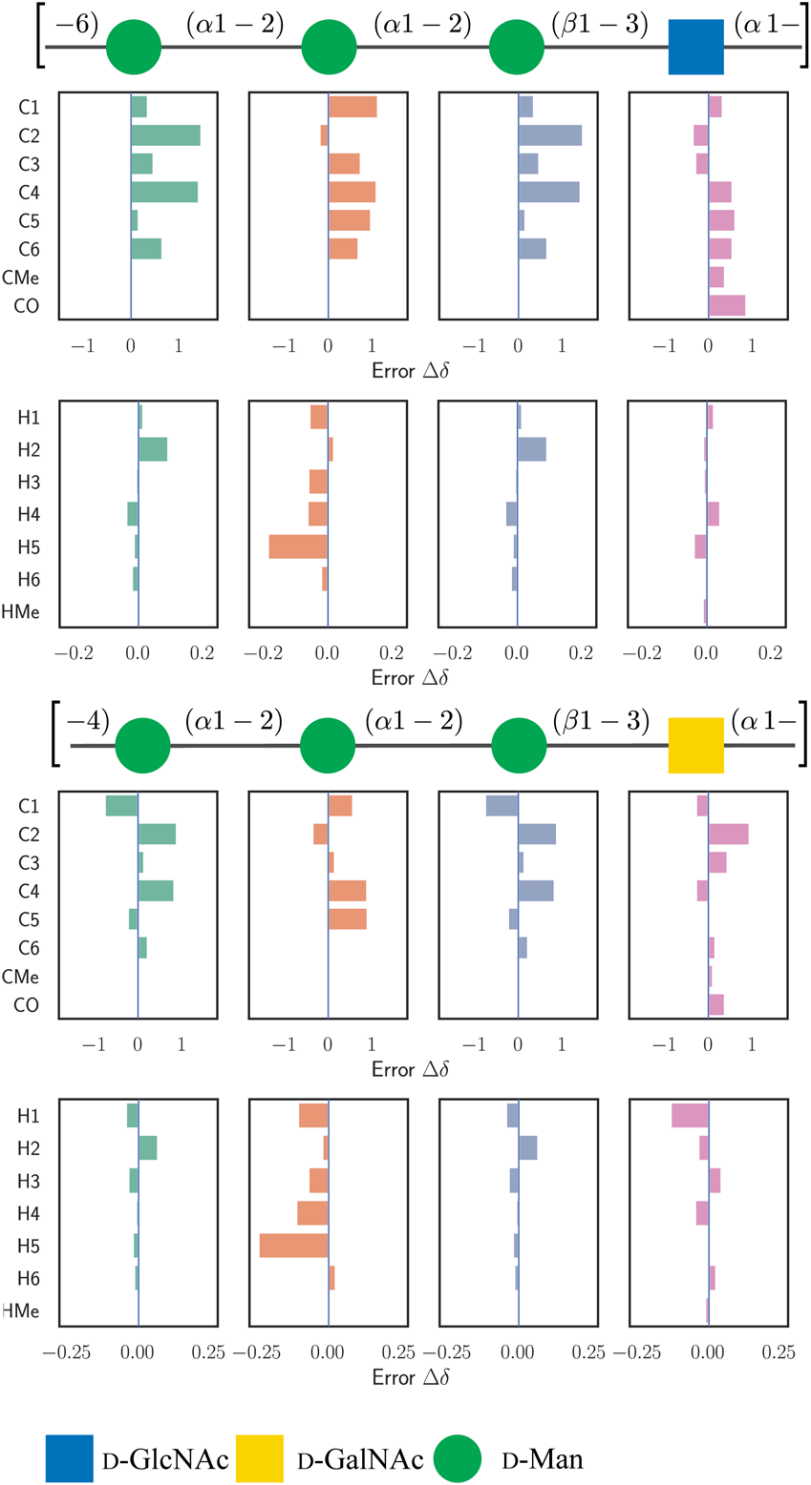
The figure illustrates the prediction errors for the ^13^C and ^1^H chemical shifts of two *E. coli* O-antigen polysaccharides, each composed of tetrasaccharide repeating units, from serogroup O77 (upper) and serogroup O176 (lower).^27,28^ The structures are visualized using symbols from the SNFG standard.^29^ The repeating units are enclosed in square brackets. The box plots visually represent the prediction errors Δ*δ* per-atom basis.

In addition to predicting the mono-, di- and trisaccharides in the original dataset, we examine GeqShift's capability to extend to larger carbohydrate structures. We predict the chemical shifts of two polysaccharides, each constructed of tetrasaccharide repeating units. In Fig. 9, the prediction accuracy of GeqShift is compared to GeqShift_inv and SG-IMP-IR. Notably, GeqShift outperforms these models regarding both ^13^C and ^1^H prediction accuracy. Furthermore, Fig. 10 details the prediction errors using bar plots for individual ^13^C and ^1^H nuclei.

## 3 Discussion

This work introduces a novel machine learning model to predict chemical shifts, explicitly addressing the stereochemistry of the molecule. We employed an Euclidean graph neural network that utilizes molecular structure and geometry as input to construct a model capable of capturing changes in molecule geometry in response to stereochemical alterations.

To enhance accuracy, we employed data augmentation techniques that replicate the dynamic behavior of molecules. Instead of restricting each molecule to a single conformation, we utilized an ensemble of conformations for both the training and testing datasets. To sample the conformations, we prioritized simplicity and speed. Therefore, we opted for the RDKit open-source toolkit, which employs an energy force field technique (further details in Section 4.3). The results in Table 2 illustrate this approach, demonstrating a decrease in mean absolute error from 0.55 to 0.34 for the predicted ^13^C chemical shifts of monosaccharides when transitioning from training the model with one conformation per molecule to training on 100 conformations per molecule.

As previously mentioned, this enhancement likely stems from two factors: a better representation of molecular reality and reduced sensitivity of the trained model to minor input variations. Relying solely on a single conformation, as done in previous attempts using 3D information in the input,^16,17^ for training poses a problem, as it leads to a less resilient model. Moreover, discovering a low-energy conformation through Density Functional Theory (DFT) is time-consuming and computationally intensive.

Because the training set includes various conformations, the model can make precise predictions when the input conformation is relatively similar to the correct one. However, there is room for improvement in conformation sampling. One potential approach for future research is to refine sampling techniques, such as those based on Gibbs free energy.

The obtained prediction errors exceeded our expectations. It must be emphasized that the ranges of chemical shifts are approximately 0–200 ppm for ^13^C and 0–10 ppm for ^1^H, so the achieved prediction errors approach the levels that qualify as error margins in measurements. However, for even better chemical shift predictions, additional developments, *e.g.*, considering the temperature at which the NMR data are acquired, will be required to evaluate and train the GNN. To further put the results into perspective, one can compare the prediction errors to other works using similar techniques for different classes of compounds and alternative ways of calculating chemical shifts. The main results are those detailed in Table 2, where our model is compared to a state-of-the-art neural network for chemical shift prediction, which has been retrained on our dataset.

The developed model has great potential for predicting chemical shifts for other organic molecules, particularly compounds with asymmetric centers. This includes, among many different classes, pharmacological compounds and proteins.

Furthermore, the ability of the model to accurately predict physical observables, *i.e.*, chemical shifts based on the molecular structure, highly encourages future application of similar methodology for other analytical techniques, *e.g.*, X-ray photoelectron spectroscopy and X-ray absorption spectroscopy and potentially for predicting other physical parameters.

Most, if not all, studies of prediction methods for NMR chemical shifts are focused on predicting chemical shifts from molecular structure. The inverse problem, where a molecular structure is generated from chemical shifts, is more compelling for experimental practice. At the same time, it is more complex. However, making proper chemical shift predictions builds a solid ground for tackling the inverse problem and a natural segue for future research. The implications are far-reaching and go beyond an advanced understanding of carbohydrate structures and spectral interpretation. For example, it could accelerate research in pharmaceutical applications, biochemistry, and structural biology, offering a faster and more reliable analysis of molecular structures. Furthermore, our approach is a key step towards a new data-driven era in spectroscopy, potentially influencing spectroscopic techniques beyond NMR.

## 4 Method

In this section, we detail the model and the dataset by giving relevant background information, then explaining GeqShift in more detail, and finally describing the carbohydrate dataset.

### 4.1 Background

#### 4.1.1 Graph neural network

A graph 
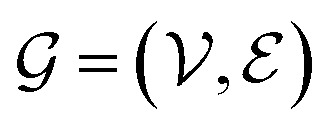
 consists of nodes 
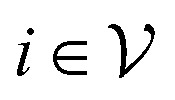
 and edges 
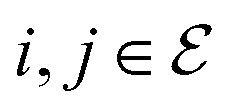
, defining the relationships between the nodes *i* and *j*. One can represent a molecule as a graph with the atoms as nodes and bonds as edges. To expand this to an even richer representation of the molecule, one can include additional edges between atoms close to each other in space; typically, we define a cutoff radius *r*_cut_ and introduce edges between any two atoms that are less than the cutoff distance apart. A graph neural network consists of multiple message-passing layers. Given a node feature **x**_*i*_^*k*^ at node *i* and edge features **e**_*ij*_^*k*^ between node *i* and its neighbors 
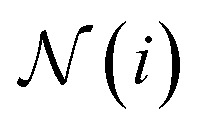
, the message passing procedure at layer *k* is defined as

1 ***m***_*ij*_^*k*^ = *f*^*m*^(**x**_*i*_^*k*^,**x**_*j*_^*k*^,**e**_*ij*_^*k*^),

2
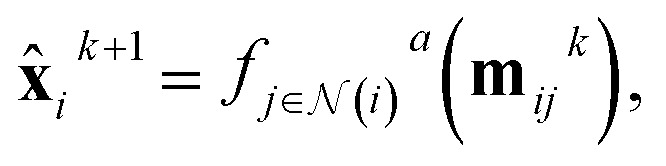

3 **x**_i_^*k*+1^ = *f*^*u*^(**x**_*i*_^*k*^,**x̂**_*i*_^*k*+1^),

where *f*^*m*^ is the message function, deriving the message from node *j* to node *i*, and 
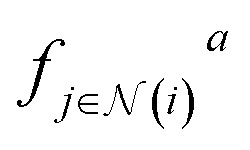
 is the aggregating function, which aggregates all messages coming from the neighbors of node *i*, defined by 
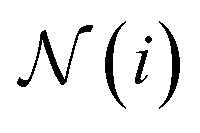
. The aggregation function is commonly just a simple summation or average. Finally, *f*^*u*^ is the update function that updates the features for each node. A graph neural network (GNN) consists of message-passing layers stacked onto each other, where the node output from one layer is the input of the successive layer.

#### 4.1.2 Equivariant convolutions

Equivariance is a fundamental concept that appears throughout the natural world, governing the symmetry and behavior of physical systems, from subatomic particles to the organization of molecules in biological systems. It underpins the consistency and invariance of natural phenomena under various transformations, making it a crucial concept in the natural sciences.

Equivariance is an essential factor when considering NMR chemical shifts. In this study, we focus on predicting the isotropic part of the chemical shift tensor, denoted as *δ*_iso_, which is a scalar and remains unchanged under the Euclidean group E(3) (the group of rotation, translation, and mirroring) with respect to the input locations of the atoms. However, the actual chemical shift tensor, ***δ***, is a second-rank tensor with an antisymmetric nature (
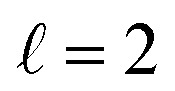
 with even parity). While it is possible to predict the complete chemical shift tensors, as demonstrated by Venetos *et al.*,^30^ molecules in solution in a laboratory setting move around relative to the external magnetic field. Consequently, it is the isotropic part of the chemical shift tensor observed in an NMR spectrum. Even though the isotropic chemical shift is a scalar quantity, the relationships governing it are intricate. Therefore, it would be advantageous to use a model capable of accurately capturing these relationships.

Euclidean neural networks can represent a comprehensive set of tensor properties and operations that obey the same symmetries as symmetries of molecules. Formally, a function *f*: *X* → *Y* is equivariant to a group of transformations *G* if for any input *x* ∈ *X* and output *y* ∈ *Y* and group element *g* ∈ *G* that is well-defined in both *X* and *Y*, we have that *fD*_*X*_(*g*)(*x*) = *D*_*Y*_(*g*)*f*(*x*), where *D*_*X*_(*g*) and *D*_*Y*_(*g*) are transformation matrices parameterized by *g* in *X* and *Y*. In other words, the result is the same regardless of whether the transformation is applied before the function or *vice versa*. An example is if you have a function deriving the interatomic forces in a molecule. These forces should be the same relative to the molecule's coordinates, independent of how the molecule is translated or rotated.

The most fundamental aspect of Euclidean neural networks involves categorizing data based on how it transforms under the operations in the Euclidean group E(3), a group in three-dimensional space that contains translations, rotations, and mirroring. These data categories are called irreducible representations (irreps) and are labeled as 
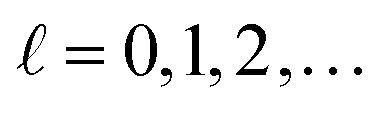
 where 
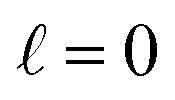
 corresponds to a scalar, while 
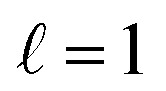
 corresponds to a three-dimensional vector. Irreps may also possess a parity, which can be either even or odd, indicating whether the representation changes signs when inverted; odd irreps change signs upon inversion, while even irreps remain unchanged. An irreducible representation with 
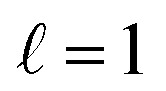
 and odd parity is termed a vector, representing entities like velocity or displacement vectors. In contrast, an irreducible representation with even parity is referred to as a pseudovector, and it characterizes properties such as angular velocity, angular momentum, and magnetic fields. The input to an Euclidean neural network is a concatenation of tensors of different data types; for example, a scalar representing a mass is concatenated with a vector representing a velocity.

We call a tensor composed of various irreducible representations a *geometric tensor*. In our graph neural network, the equivariant version of vector multiplication involves two geometric tensors and is known as a tensor product ***x*** ⊗ _***w***_***y***. Here, ***w*** are learnable weights. Our approach employs these tensor products for equivariant message passing, departing from conventional linear operations. For a more in-depth exploration of Euclidean graph neural networks, we refer readers to the study by Geiger *et al.*^31^

### 4.2 Machine learning model

We construct an equivariant graph self-attention network where the input to the network depends on the chemical structure 
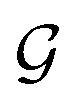
 and the atom positions matrix ***R*** of the specific molecule (see Fig. 4). We exclude hydrogen atoms from the representation of molecules to reduce computational complexity. Every atom/node is represented by a learnable embedding vector ***x***_*i*_, where the embedding depends on the specific atom type *Z*_*i*_ (for example, 4 for carbon or 8 for oxygen) and the number of hydrogen atoms connected to that particular atom Ni^*h*^. The node/atom input embedding vector is

4 **x**^0^_*i*_ = (Emb(*Z*_*i*_))‖Emb(*N*_*i*_^*h*^)),

where we denote the concatenation of two vectors with (·‖·). We create edges between all atoms in the molecule within a cutoff radius *r*_cut_ = 6 Å. Every edge is represented by a vector of scalars (
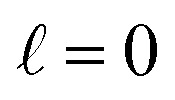
 and even parity) **h**_*ij*_^*s*^=(Emb(*E*_*ij*_)‖*d*_*ij*_) where Emb(*E*_*ij*_) is an embedding vector depending on the particular bond type *E*_*ij*_ (no bond, single bond, or double bond), and *d*_*ij*_ = ‖**r**_*i*_ – **r**_*j*_‖ is the euclidean distance between the nodes *i* and *j*. We also construct an embedding of the normalized relative distance between the nodes/atoms, **r̂**_*ij*_ = **r**_*i*_ − **r**_*j*_ using spherical harmonics 
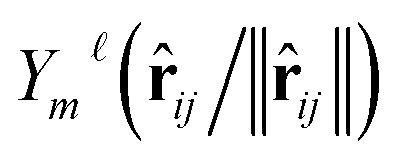
, where *m* is the parity and 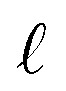 is the rotation order.

The layers in the network consist of E(3)-equivariant self-attention/transformer layers,^22,32^ built using the *e3nn* library.^31^ For the layers *k* = 1, …, *K*, we derive messages by deriving queries ***q***^*k*^, keys ***k***^*k*^, and value ***v***^*k*^ as

5 **q**_*i*_^*k*^ = Linear(**x**_*i*_^*k*^)

6
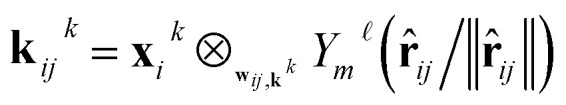
7
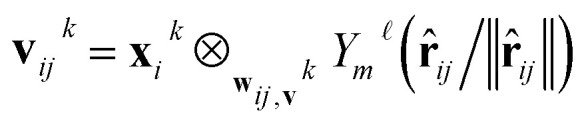
where linear is a generalization of a regular linear layer for a geometric tensor. The weights of the tensor products ⊗ are derived by neural networks, with the invariant edge embeddings as inputs: *w*_*ij*_^*k*^ = NN_*k*_(*e*_*ij*_^*s*^) and *w*_*ij*_^*v*^ = NN_*v*_(*e*_*ij*_^*s*^). The self-attention is derived as8
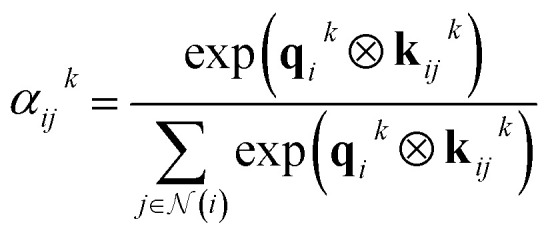
where ***q***_*i*_⊗***k***_*ij*_ maps to a scalar 
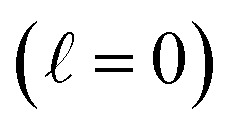
. We aggregate the messages by summing up the weighted messages from all neighboring nodes 
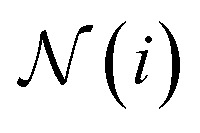
9
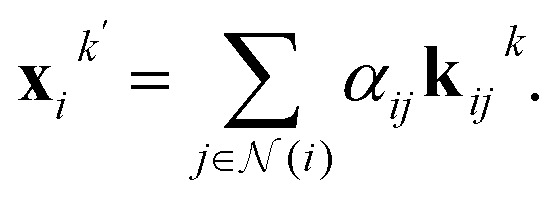


In between the self-attention layers, the geometric tensors are updated with equivariant Layer Normalization (LN)^22^ and an equivariant neural network (NN) as

10 *x*_*i*_^*k*+1^ = LN(NN(*x*_i_^*k*′^) + *x*_*i*_^*k*^),

where the neural network consists of the generalized linear layers (Linear) and Sigmoid linear units (SiLU) activation functions. The last layer *K* output is an invariant vector **x**_*i*_^*K*^. Finally, a multilayer perceptron with scalar output is applied.

We train the model by minimizing the mean absolute error,11
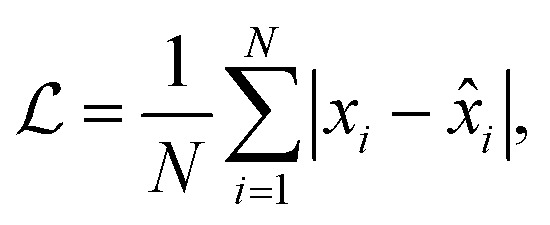
where *N* is the number of chemical shifts, *x*_*i*_ is the experimentally determined chemical shift, and *x̂*_*i*_ is the predicted one.

We train the model with multiple conformations and, thereby, multiple graphs for each chemical shift *x*_*i*_. This results in an ensemble of predictions *x̂*^0^_i_,…,*x̂*_*i*_^*j*^,*x̂*_*i*_^*N*_*i*_^ for every output *x*_*i*_. We want the average of this ensemble to be equal to the experimentally determined chemical shift, such that 
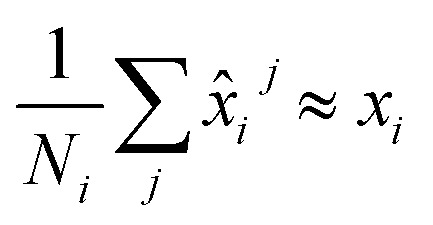
. Thus, we aim at minimizing 
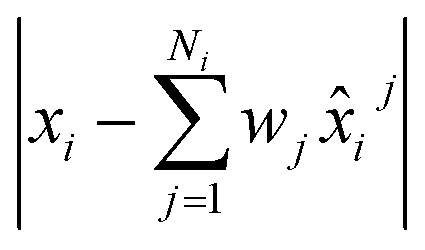
. It follows from the triangle inequality that12
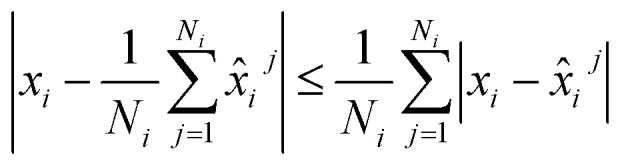
hence, we can minimize the right-hand side of the eqn (12). This results in the relatively simple conclusion that we, in the training dataset, can add the ensemble of conformations to create a single large training dataset.

#### 4.2.1 Implementation details

The dimension of the input node embedding **x**^0^_*i*_ is 128, and the input scalar edge embedding **e**^0^_*ij*_ is 32. The model consists of seven layers where the hidden dimensions between the layers consist of a scalar vector of size 64, 32 tensors with 
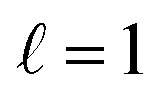
 and odd parity, and eight tensors with 
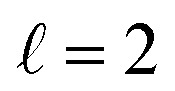
 and even parity. Between the self-attention layers, the hidden layer is passed through an equivariant neural network with one hidden layer and a SiLU non-linearity, followed by an equivariant layer normalization. The last layers map the tensors to a scalar vector with 128 dimensions. This vector is passed through a two-layer multilayer perceptron with a hidden dimension 384 and an output dimension of one.

The batch size of the models is set to 32 except for SG-IMP-IR, where the recommended batch size of 128 is used. The models are optimized using the Adam optimizer^33^ starting with a learning rate of 3 × 10^−4^. We used a small validation set of five percent of the training data for the models trained using only one conformation per molecule. The learning rate decreased during training using the PyTorch ReduceLROnPlateau, which reduces the error when the validation error stops decreasing. A patience of 20 epochs and a reducing factor of 0.1 was used. We did not use a scheduler for instances when multiple conformations were used. Instead, we trained these models during three epochs, and the learning rate decreases by 0.1 for every new epoch.

The model is implemented using Python 3.9.13, PyTorch version 2.0.0, Cuda version 11.7, PyTorch geometric version 2.3.0, e3nn version 0.5.1, RDKit version 2022.09.5, and GlyLES version 0.5.11. The models are trained using one NVIDIA A100 GPU. The training time per model takes around 30 minutes to an hour.

### 4.3 The dataset

The dataset consists of experimental data of ^1^H and ^13^C NMR chemical shifts of mono-to trisaccharides. The data is used by CASPER^7,34,35^ and is based on published data http://www.casper.organ.su.se/casper/liter.php, including, *inter alia*, those related to structures of biological interest.^36–38^ In detail, it encompasses ^1^H and ^13^C NMR chemical shifts for 375 carbohydrates in aqueous solution. Of these are 107 monosaccharides, 153 disaccharides, and 115 trisaccharides. By summing up the individual shifts, the dataset contains 5356 ^1^H and 4713 ^13^C chemical shifts. *GlyLES*^39^ was used to convert the carbohydrates from the IUPAC representation into SMILES representation. The open-source library^40^ was used to convert the molecule from the SMILES representation to a graph. RDKit was also used to generate molecular conformations. To obtain 100 conformations per molecule, we generated 200 conformations using the ETKDGv3 method.^41^ To gain a spread in the conformational distribution, we enforced keeping only conformations at a certain distance from each other; the RMSD between the heavy atoms is larger than 0.01 Å. By deriving the potential energy using the MMFF94 force field,^42^ we discarded the 100 conformations with the highest energy.

The CSDB predictions are simulated at http://csdb.glycoscience.ru/. The NMR spectrum assignment was done with the help of the chemical shift reference collection and simulation tool for ^13^C^43,44^ and ^1^H^44^ nuclei at the Carbohydrate Structure Database (CSDB).^45^ To refine a set of structural hypotheses, the CSDB structural ranking tool^46^ and empirical chemical shift simulation^47^ were used. We use the hybrid carbon chemical shift simulation.

The NMRDB predictions for ^13^C^48–50^ and ^1^H^48,49,51^ are simulated at https://www.nmrdb.org/.

## Code availability

The code is available at https://github.com/mariabankestad/GeqShift.

## Data availability

The dataset of ^1^H and ^13^C NMR chemical shifts are available at https://github.com/mariabankestad/GeqShift.

## Author contributions

M. B. developed and implemented the software, conducted the experiments, and produced the illustrations. J. R., K. D., and G. W. contributed with the data and with expertise in carbohydrates. All four authors M. B, J. R, K. D and G. W. took part in writing the manuscript.

## Conflicts of interest

There are no conflicts to declare.
